# Associating dose–volume characteristics with theoretical radiobiological metrics for rapid Gamma Knife stereotactic radiosurgery plan evaluation

**DOI:** 10.1002/acm2.13018

**Published:** 2020-09-10

**Authors:** Christopher J. Tien, James E. Bond, Zhe (Jay) Chen

**Affiliations:** ^1^ Department of Therapeutic Radiology Yale School of Medicine New Haven CT USA

**Keywords:** dose–volume histogram, equivalent uniform dose, Gamma Knife, radiosurgery, tumor control probability

## Abstract

**Purpose:**

To examine general dose–volume characteristics in Gamma Knife (GK) plans which may be associated with higher tumor control probability (TCP) and equivalent uniform dose (EUD) using characteristic curve sets.

**Methods:**

Two sets of dose–volume histograms (DVHs) were exported alongside an analytical purpose‐generated DVH: (a) single‐shot large collimator (8 or 16 mm) emulated with multiple shots of 4 mm collimator. (b) shot‐within‐shot (SWS) technique with isodose lines (IDLs) of 40–75%. TCP, average dose, EUD in single‐fraction (EUD_T_) and 2 Gy fractionated regimens (EUD_R_) were examined for trends with cumulative DVH (cDVH) shape as calculated using a linear‐quadratic cell survival model (α/β = 10.0 Gy, N_0_ = 1 × 10^6^) with both α = 0.20 Gy^−1^ and α = 0.23 Gy^−1^.

**Results:**

Using α = 0.20 Gy^−1^ (α = 0.23 Gy^−1^), plans in the analytical set with higher shoulder regions had TCP, EUD_T_, EUD_R_ increased by 180%, 5.9%, 10.7% (11.2%, 6.3%, 10.0%), respectively. With α = 0.20 Gy^−1^ (α = 0.23 Gy^−1^), plans with higher heels had TCP, EUD_T_, EUD_R_ increased by 4.0%, <1%, <1% (0.6%, <1%, <1%), respectively. In emulating a 16 (8) mm collimator, 64 (12) shots of the small collimators were used. Plans based on small collimators had higher shoulder regions and, with α = 0.20 Gy^−1^ (α = 0.23 Gy^−1^), TCP, EUD_T_, EUD_R_ was increased up to 351.4%, 5.0%, 8.8% (270.4%, 5.0%, 6.8%) compared with the single‐shot large collimator. Delivery times ranged from 10.2 to 130.3 min. The SWS technique used 16:8 mm collimator weightings ranging from 1:2 to 9.2:1 for 40–75% IDL. With α = 0.20 Gy^−1^ (α = 0.23 Gy^−1^), the 40% IDL plan had the highest shoulder with increased TCP, EUD_T_, EUD_R_ by 130.7%, 9.6%, 17.1% (12.9%, 9.1%, 16.4%) over the 75% IDL plan. Delivery times ranged 6.9–13.8 min.

**Conclusions:**

The magnitude of the shoulder region characteristic to GK cDVHs may be used to rapidly identify superior plan among candidates. Practical issues such as delivery time may require further consideration.

## 1 INTRODUCTION

Gamma Knife (GK) radiosurgery treatment plans are unique in their low (typically ~ 50%) prescription isodose lines,^1^, ^2^ which leads to a highly heterogeneous dose distribution within the target. Given that portions of the target will receive doses up to twice as high as the prescription dose, along with the versatility of the GK collimator delivery system, vastly different dose distributions can be generated depending on the planning team. This, in addition to dose ranges which are typically at least 15–18 Gy in a single fraction,^1^, ^2^, ^3^ results in a persistent quest in GK treatment planning on identifying favorable dosimetric characteristics of GK plans that would produce the best tumor control is acheived.^4^

Common plan quality indices such as the conformity index^1^, ^5^ or the Paddick index^6^ are focused upon the shape and coverage of the prescription isodose. These indices are convenient, as simple ratios of relevant volumes, and can be calculated with minimal additional training for the treatment planning team. In exchange for its simplicity, however, these indices do not account for the shape and/or coverage of other isodose lines enclosed within the prescription isodose volume (PIV), which could also affect the clinical effectiveness of the GK plan. Dose–volume traits within the target are a source of raw data which could be used to compute a more clinically relevant objective measure of the plan quality.

Two metrics which can capture the complete dose–volume characteristics of the target are the tumor control probability (TCP) and equivalent uniform dose (EUD). Both metrics provide an objective quantification of plan quality through comprehensive dose–volume characteristics. Furthermore, these metrics also correlate with an intuitive interpretation — TCP represents a straightforward biological endpoint and EUD aids in reframing a dose distribution as a more familiar uniform dose distribution. However, these metrics require additional calculations not readily available within the current GK treatment planning software.

Since ideal dose‐volume histograms (DVHs) should inherently possess some general shapes and/or visual features that maximize the plan quality, the aim of this study was to examine and identify favorable dose‐volume histogram characteristics or features of GK plans that would result in the highest TCP and EUD.^4^ If it is indeed possible to uncover favorable shapes and/or visual features within a dose‐volume histogram, this could be incorporated by the treatment planning team to rapidly identify the most robust plan from a cohort of candidate plans.

## 2 MATERIALS AND METHODS

### 2.A Radiobiological analysis

Our investigation is based on the linear‐quadratic (LQ) cell survival model. In general, the utility of the LQ model is predicated on its simplicity in only using a handful of parameters. Thus, the resultant TCP is especially sensitive to values chosen for its radiobiologic parameters: α, α/β, and N_0_. It is well‐known that, due to the interpatient heterogeneity, no single set of parameters is feasible for any patient population.^7^

With this in mind, we selected the two sets of radiobiological parameters. The first set was designed to exaggerate differences in cell survival fraction by assigning a lower α value — α = 0.20 Gy^−1^, α/β = 10.0 Gy. Parameters were selected within the sharp gradient region of the sigmoidal TCP curve to increase sensitivity to uncover trends.

The second set was a more clinically realistic parameter set which was calibrated to provide more familiar TCP results by assigning a higher α value: α = 0.23 Gy^−1^, α/β = 10.0 Gy. These parameters lie in the flatter plateau region of the TCP sigmoidal distribution.

The LQ cell survival model calculates the tumor cell survival fraction, S, for each plan, assuming a homogeneous initial tumor cell population as

(1) $$S=\sum_{j=1}^{J}dDVH\left(D_{j}\right)\Delta De^{‐\alpha D_{j}‐\beta D_{j}^{2}}$$

where α and β are radiobiological parameters of the LQ model, dDVH(D_j_) is the differential DVH value associated with dose D_j_, and ΔD is the dose increment in the DVH. Tumor control probability (TCP) was calculated assuming a Poisson distribution as

(2) $$TCP=e^{‐N_{0}S}$$

where N_0_ was the initial number of tumor cells before irradiation, and was assigned a value of N_0_ = 1 × 10^6^ for both datasets. The equivalent uniform dose (EUD) for a single‐fraction (EUD_T_) was calculated as^8^

(3) $$EUD_{T}=\frac{1}{D_{\mathrm{ref}}}\left(‐\frac{\alpha }{\beta }+\sqrt{{\left(\frac{\alpha }{\beta }\right)}^{2}+4D_{\mathrm{ref}}\left(\frac{\alpha }{\beta }+2\right)\frac{\ln\left(S\right)}{\ln\left(SF_{2}\right)}}\right)$$

where SF_2_ was the survival fraction applied with a single 2 Gy irradiation and D_ref_ was the reference dose per fraction (2 Gy). The corresponding EUD for a fractionated regimen in 2 Gy fractions was calculated as^8^

(4) $$EUD_{R}=EUD_{T}\frac{\alpha /\beta +EUD_{T}}{\alpha /\beta +2}$$

### 2.B **Analytical purpose‐generated dose**‐**volume‐histograms**

To aid systematic investigation of the influence of DVH shapes on TCP or EUD, a realistic set of dDVHs similar to those observed in GK SRS cases was generated using a custom set of equations proposed by Chen et al.^4^ Each dDVH was subsequently converted to a cumulative DVH (cDVH). The resultant analytical cDVH was composed of six regions. The width of each region was based on parameters including minimum dose (D_min_), maximum dose (D_max_), and three inflection points (D_1_, D_mid_, D_2_). The degree of curvature was defined using tunable parameters of h_1_, h_2_ in addition to the values of dose regions (D_min_, D_max_, D_1_, D_mid_, D_2_). Dose was normalized to a maximum dose with 201 dose bins.

The equations used to generate the dDVH curves are shown below, where each curve was defined by

(5) $$C_{1}=h_{1}C_{\mathrm{mid}}$$

(6) $$C_{2}=h_{2}C_{\mathrm{mid}}$$

(7) $$C_{\mathrm{mid}}=\frac{2}{C_{1}\left(D_{\mathrm{mid}}‐D_{\min}\right)+C_{2}\left(D_{\max}‐D_{\min}\right)+D_{2}‐D_{1}}$$

(8) $$dDVH\left(D\right)=\left\{\begin{array}{cc} 0, & D<D_{\min}\\ C_{1}\frac{D‐D_{\min}}{D_{1}‐D_{\min}}, & D_{\min}<D<D_{1}\\ C_{1}+\left(C_{\mathrm{mid}}‐C_{1}\right)\frac{D‐D_{1}}{D_{\mathrm{mid}}‐D_{1}}, & D_{1}<D<D_{\mathrm{mid}}\\ C_{\mathrm{mid}}+\left(C_{2}‐C_{\mathrm{mid}}\right)\frac{D‐D_{\mathrm{mid}}}{D_{2}‐D_{\mathrm{mid}}}, & D_{\mathrm{mid}}<D<D_{2}\\ C_{2}‐C_{2}\frac{D‐D_{2}}{D_{\max}‐D_{2}}, & D_{2}<D<D_{\max}\\ 0, & D>D_{\max} \end{array}\right)$$

Prescription dose was set as 18 Gy at the 50% IDL for all plans, which is our institution’s most common value. Thus, D_min_ and D_max_ were set at 18 and 36 Gy, respectively, in Eqs. ((5), (6), (7), (8)).

### 2.C Single‐shot large collimator emulated with multiple shots of small collimator

The second set of DVH curves was generated by comparing single‐shot against multiple‐shot distributions for both large collimator sizes (8 and 16 mm). For each of the large collimators, this technique was designed to produce two plans with an indistinguishable prescription isodose volume (PIV) but distinctly different dose distribution elsewhere.

For the single‐shot distribution, the 50% IDL surface of a single‐shot large (8 or 16 mm) collimator was converted into a target contour (“large‐8” or “large‐16,” respectively). The complementing multiple‐shot distribution was created using exclusively 4 mm collimator shots with multiple isocenters to mimic the reference contour (large‐8 or large‐16) created using single‐shot of the large collimator. Each plan was prescribed 18 Gy at the 50% IDL.

### 2.D Nested shot‐within‐shot (SWS) technique

The third set of DVH curves was generated using the shot‐within‐shot (SWS) planning technique. This method pairs the 8mm collimator with either a 4mm or 16mm collimator weighted to produce a 50% IDL diameter of 4–8 mm or 8–16 mm, respectively. Simultaneously, an appropriate isodose line was selected to scale the absolute dose to the target. Prior studies have shown the efficacy of this method of pairing shot weighting with IDL to generate the same absolute dose to the prescription IDL.^9^, ^10^

Prescription isodose lines of 40%, 45%, 50%, 55%, 60%, 65%, 70%, and 75% were each assigned to different plans using a 16 to 8mm SWS pair. For each isodose line, the weighting was adjusted to match the absolute prescription (18 Gy) to the prescription IDL such that the PIV was identical to the reference plan — the 50% IDL of an evenly weighted 1:1 plan. For the dose–volume analysis, the target volume GTV was defined as the PIV of the reference 1:1 plan minus a 1mm margin.

## 3 RESULTS

### 3.A Purpose‐generated analytical histograms

**Fig. 1:**
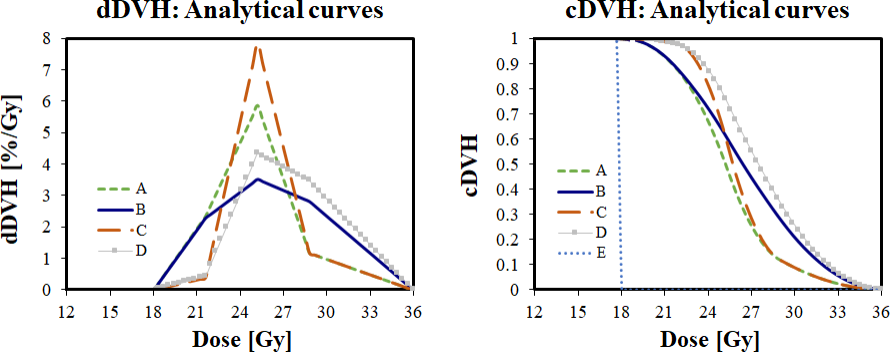
Corresponding differential dose‐volume histograms (dDVH)(left) and cumulative DVH (cDVH) (right) generated using Eqs. ((1), (2), (3), (4)). Note, curve E has a single value of dDVH = 100% at 18 Gy in the dDVH and is not shown due to scaling. For all curves, prescription dose was set to 18 Gy at 50% isodose line. The values for D_1_, D_2_, D_mid_, h_1_, h_2_ are summarized in Table A1.

A set of five dDVH curves was created with accompanying cDVH curves, which are shown together in Fig. 1. For the first four curves (A, B, C, D), D_min_ and D_max_ were set at 18.0 and 36.0, respectively. Using Eqs. (1)–(4), values were selected for D_1_, D_2,_ D_mid_, h_1_, h_2_ (summarized in Table A1) and used to derive remaining parameters: C_1_, C_2,_ C_mid_. For these curves, values were chosen to create distinct, yet paired variations in each curve for the shoulder and fall‐off regions.

The last curve, E is a Dirac step function in the cDVH, created by setting the dDVH to 100.0 at 18.0 Gy. This was performed manually rather than using the analytical equations to avoid dividing by zero for curve.

**Fig. 2:**
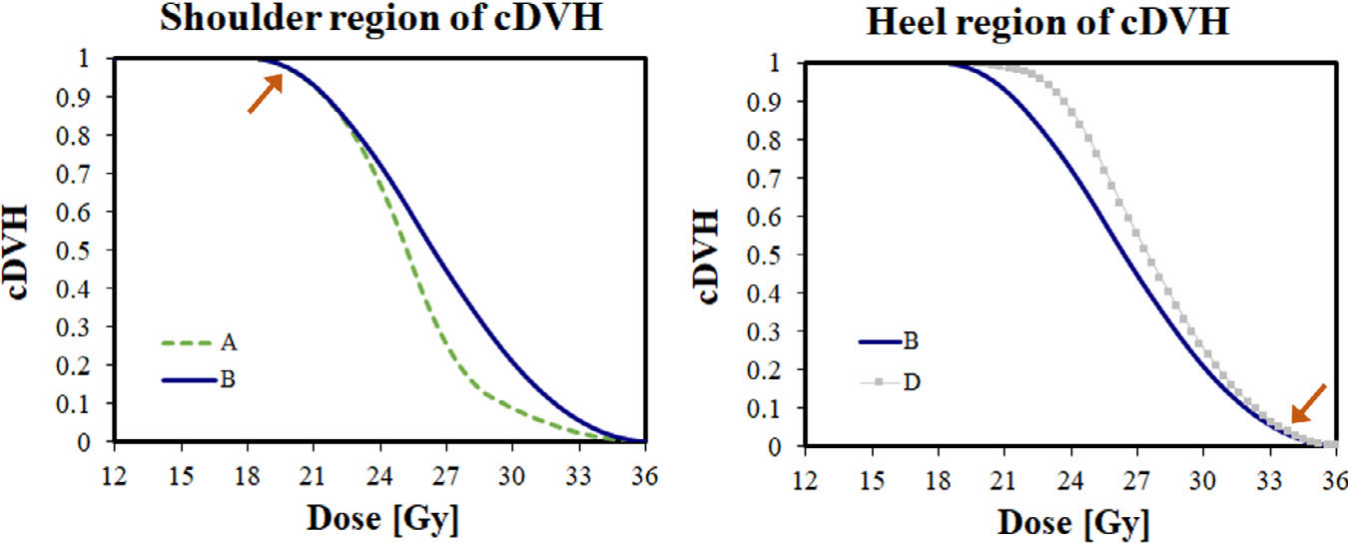
Cumulative dose‐volume histogram (cDVH) for curves A and B (left) show similar shoulder region (see arrow), while curves B and D (right) show similar heel region (see arrow).

The “shoulder” and “heel” of the cDVH regions are shown in Fig. 2. Shoulder (D_90%_‐D_95%_ region) and heel (D_5%_–D_10%_ region) attributes between curves were purposely manipulated to create similar pairings of low and high attributes for comparison. For example, Curves A and B have similar shoulder attributes, but varying heel attributes (see Fig. 2). Similarly, Curves B and D have similar heel attributes, but varying shoulder attributes (see Fig. 2).

Survival fraction for 2 Gy irradiations (SF_2_) was 0.619 and 0.576 for α = 0.20 Gy^−1^ and α = 0.23 Gy^−1^, respectively. D_avg_, EUD, and TCP for both values of α are shown in Table 1. The D_avg_ ranged between 25.2 and 27.5 Gy. For both parameter sets, the curve with the lowest D_avg_ (curve E) had the lowest associated TCP, and the curve with the highest D_avg_ (curve D) had the highest associated TCP. However, the other curves (A, B, C) did not demonstrate a monotonic relationship between D_avg_ and TCP.

**Table 1:** Average dose, equivalent uniform dose (EUD), and tumor control probability (TCP) for analytically generated cumulative dose‐volume histogram (cDVH) curves with α = 0.20 Gy^−1^ and α = 0.23 Gy^−1^. Each curve had α/β = 10.0 Gy, and N_0_ = 1 × 10^6^ and a different combination of shoulder and heel attributes. Prescription dose was set as 18 Gy at 50% isodose line.

Curve	Shoulder	Heel	D_avg_ [Gy]	α = 0.20 Gy^‐1^, α/β = 10 Gy, N_0_ = 1 × 10^6^			α = 0.23 Gy^‐1^, α/β = 10 Gy, N_0_ = 1 × 10^6^		
				EUD_T_ [Gy]	EUD_R_ [Gy]	TCP [%]	EUD_T_ [Gy]	EUD_R_ [Gy]	TCP [%]
A	Low	Low	25.2	22.0	58.5	45.2	21.7	57.4	87.5
B	Low	High	26.4	22.0	58.7	47.0	21.7	57.5	88.0
C	High	Low	25.9	23.3	64.7	83.5	23.0	63.1	97.3
D	High	High	27.5	23.4	65.0	84.4	23.0	63.2	97.3
E	–	–	18.0	18.0	42.0	0	18.0	42.0	0

Abbreviations: PIV, prescription isodose volume; D_avg_, average dose; EUD_T_, equivalent uniform dose for a single‐fraction; EUD_R_, equivalent uniform dose for a fractionated 2 Gy regimen; TCP, tumor control probability.

For α = 0.20 Gy^−1^, curves A and B provided similar shoulder regions in the cDVH immediately above prescription dose, while varying the heel region. The increase in cDVH values in the heel region resulted in <2% improvement in TCP, from 45.2% to 47.0%. Similarly, curves C and D had similar shoulders, while varying the heel regions. The increase in the cDVH heel region resulted in <1% improvement in TCP, from 83.5% to 84.4%. Both the EUD_T_ and EUD_R_ followed the same trend: for curves with similar shoulder regions, slight increases for higher heel regions, resulted in a small increase (<1%) .

For α = 0.20 Gy^−1^, curves A and C provided similar heel regions in the cDVH near maximum dose, while varying the shoulder region. The increase in values in the cDVH shoulder region nearly doubled (185%) TCP from 45.2% to 83.5%. Similarly, curves B and D provided similar heel regions in the cDVH near maximum dose, while varying the shoulder region. The increase in the cDVH shoulder region increased TCP 180%, from 47.0% to 84.4%. The EUD_T_ and EUD_R_ increased by 6.3% and 10.7%, respectively.

Using a different radiobiological parameter set with α = 0.23 Gy^−1^, the same overall trends were observed to a milder degree: for curves with similar heels, increasing the shoulder region yielded considerably higher (11.2%) TCP than the heel region (0.6%). With the larger α value, the overall TCP values were much larger. While the increase in TCP were milder, the EUD_T_ and EUD_R_ were slightly higher, with increases of 6.3% and 10.0%, respectively.

### 3.B Single‐shot large collimator emulated with multiple shots of small collimator

The prescription IDL coverage of the single‐shot 16 and 8mm collimators nominally define a PIV of 16 and 8 mm, respectively. For single‐shot plans, the isodose lines were concentric circles with dose monotonically increasing as radial distance from isocenter decreased.

Using 4 mm collimators, 64 and 12 shots with multiple isocenters were used to reproduce the large collimator PIVs. Contrary to the single‐shot plans, the multiple‐shot plans were heterogeneous with a series of symmetric local minimums and maximums. The dose profile undulated with radial distance from isocenter. The dose at the isocenter was 23.0 and 33.8 Gy for the 16 and 8mm PIV, respectively. The maximum dose regions were located 5.1 and 1.8 cm radially from the isocenter for the 16mm and 8mm PIV.

As shown in Table 2, due to the large number of shots required for the 4mm collimators, matching the 16 and 8mm PIV collimators required delivery times of 130.3 and 42.8 min, respectively (scaled down to match the 3.5 Gy/min dose rate immediately following source‐exchange), for 18 Gy at the 50% IDL. These delivery times were about an order of magnitude larger than single‐shot deliveries using 16 and 8 mm collimators, which required 10.2 and 11.4 min, respectively. The longer delivery times for the 4mm collimator were mainly due to the sheer number of shots required, and was also amplified by its lower output factor (0.814).

**Table 2:** Average dose, equivalent uniform dose (EUD), and tumor control probability (TCP) for analytically generated cumulative dose‐volume histogram (cDVH) curves with α = 0.20 Gy^‐1^ and α = 0.23 Gy^‐1^. Each with α/β = 10.0 Gy, and N_0_ = 1 × 10^6^ for curves created by using 64 or 12 4 mm collimators to emulate 16 or 8 mm collimators, respectively. Prescription dose was set as 18 Gy at 50% IDL with no margin on the PIV.

Plan ID	PIV diameter [mm]	Collimators used		Delivery time [min]^a^	D_avg_ [Gy]	α = 0.20 Gy^‐1^, α/β = 10 Gy, N_0_ = 1 × 10^6^			α = 0.23 Gy^‐1^, α/β = 10 Gy, N_0_ = 1 × 10^6^		
		Size	#			EUD_T_ [Gy]	EUD_R_ [Gy]	TCP [%]	EUD_T_ [Gy]	EUD_R_ [Gy]	TCP [%]
Plan16_16	16	16	1	10.2	28.7	22.1	59.2	10.9	21.9	58.0	65.5
Plan16_4	16	4	64	130.3	26.5	23.2	64.4	49.2	23.0	63.1	88.9
Plan8_8	8	8	1	11.4	27.4	22.1	59.2	10.8	21.9	58.0	65.5
Plan8_4	8	4	12	42.8	26.8	23.0	63.2	40.0	22.7	62.1	85.9

Abbreviations: PIV, prescription isodose volume; D_avg_, average dose; EUD_T_, equivalent uniform dose for a single‐fraction; EUD_R_, equivalent uniform dose for a fractionated 2 Gy regimen; TCP, tumor control probability.

^a^ Delivery time scaled to nominal dose rate of 3.5 Gy/min.

**Fig. 3:**
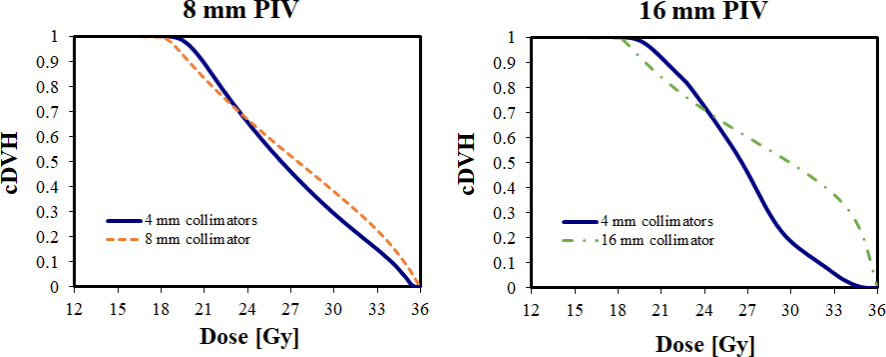
Cumulative dose‐volume histogram (cDVH) for a 16mm (left) and 8mm (right) prescription isodose volume (PIV), covered using a single‐shot using large collimator or with multiple shots of the 4mm collimator.

The cDVHs are shown for a 16 and 8mm PIV in Fig. 3. Geometrically, using smaller 4mm collimator shots resulted in a dose distribution with a large ring of shots to cover the periphery of the PIV. Compared to the larger single‐shot collimators, the 4mm collimator shot dose distributions demonstrated three trends. First, the region of maximum dose was drawn from a point dose at the center of the target into an annular ring a few cm (1.8–5.1 cm) away. Second, the entire outer edge of the PIV received a higher average dose, which was reflected in a larger cDVH shoulder. Third, the interior volume of the PIV received a lower average dose, which was reflected in a lower cDVH heel.

As summarized in Table 2, for α = 0.20 Gy^−1^, there was a significant improvement in the TCP when using the 4mm collimators, with around fourfold increase seen in both 16mm (451%) and 8mm (370%) collimators. The EUD_T_ increased by 5.0% and 4.1% for the 16 and 8 mm collimators, respectively. The EUD_R_ increased by 8.8% and 6.8% for the 16 and 8mm collimators, respectively.

Using a different radiobiological parameter set, with α = 0.23 Gy^−1^, the same overall trends were observed, but to a milder degree; the increased shoulder of the 4mm collimator curves in the cDVH improved the TCP for both the 16mm (35%) and 8mm (31%) collimators. The EUD_T_ increased by 5.0% and 3.7% for the 16 and 8 mm collimators, respectively.The EUD_R_ increased by 8.8% and 7.1% for the 16 and 8mm collimators, respectively.

### 3.C Nested shot‐within‐shot(SWS) technique

**Fig. 4:**
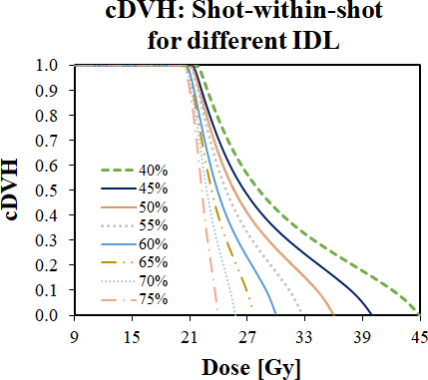
Cumulative dose‐volume histogram (cDVH) created using different isodose lines (IDLs) prescribed to different plans using the shot‐within‐shot (SWS) technique based on a pair of 16 and 8 mm collimators. Each plan was designed to create the same prescription isodose volume (PIV) as a 1:1 (16:8 mm) reference weighting.

Isodose lines of 40%, 50%, 55%, 60%, 65%, 70%, and 75% were prescribed 18 Gy to various weightings of 16 and 8mm collimators. An evenly weighted 1:1 distribution prescribed to the 50% IDL was used as a reference PIV, which had a volume of 1.443 cm^3^. For cDVH values shown in Fig. 4, the target volume was defined as the reference PIV minus a 1mm margin.

As summarized in Table 3, the SWS technique was able to mimic a variety of IDLs ranging from 40% to 75%. With the appropriate weightings to match the desired IDL, the PIV of the SWS technique was visually indistinguishable from the reference pair with a 1:1 (16 and 8 mm) weighting. The maximum deviation in PIV from reference was 1.4%.

**Table 3:** Average dose, equivalent uniform dose (EUD), and tumor control probability (TCP) for different isodose lines using nested shot‐within‐shot (SWS) technique, each with α/β = 10.0 Gy and N_0_ = 1 × 10^6^, for α = 0.20 Gy^−1^ and α = 0.23 Gy^−1^.

Rx IDL	Weighting		PIV/Ref	Delivery time [min]^a^	D_avg_ [Gy]	α = 0.20 Gy^‐1^, α/β = 10 Gy, N_0_ = 1 × 10^6^			α = 0.23 Gy^‐1^, α/β = 10 Gy, N_0_ = 1 × 10^6^		
	16 mm	8 mm				EUD_T_ [Gy]	EUD_R_ [Gy]	TCP [%]	EUD_T_ [Gy]	EUD_R_ [Gy]	TCP [%]
40	1	2	0.986	13.8	30.4	25.2	74.0	91.8	25.0	73.0	99.1
45	1	1.4	0.994	12.2	28.3	24.6	70.9	84.6	24.4	70.1	98.0
50	1	1	–	10.9	26.9	24.4	69.7	80.5	24.2	69.0	97.4
55	1.4	1	1.010	9.8	25.7	24.1	68.6	75.8	24.0	68.0	96.7
60	1.9	1	0.993	8.9	24.5	23.7	66.5	64.4	23.6	65.9	94.5
65	2.8	1	0.996	8.1	23.7	23.5	65.5	57.4	23.4	65.0	93.0
70	4.5	1	0.998	7.5	22.9	23.2	64.4	49.3	23.1	63.9	91.0
75	9.2	1	1.001	6.9	22.3	23.0	63.2	39.8	22.9	62.7	87.8

Abbreviations: Rx, prescription; IDL, isodose line; PIV, prescription isodose volume; ref, reference volume; D_avg_, average dose; EUD_T_, equivalent uniform dose for a single‐fraction, EUD_R_, equivalent uniform dose for a fractionated 2 Gy regimen; TCP, tumor control probability.

^a^ Delivery time scaled to nominal dose rate of 3.5 Gy/min.

Each plan was prescribed 18 Gy and the delivery times were scaled down to match the 3.5 Gy/min dose rate immediately following source‐exchange. The global maximum dose to the PIV resulted in a significant difference in delivery time based upon the IDL and SWS pair selected. Additionally, there was a 10% reduction in output factor when using 8 mm rather than 16 mm collimators. Overall, this resulted in the 40% IDL plan requiring double (200%) the delivery time of the 75% IDL plan.

Each value of the cDVH was uniformly larger for lower IDL prescriptions. Unlike the previous section, higher shoulders were also observed with higher heels. The lower IDL plans had a higher global maximum dose, which contributed to the larger shoulders observed in the cDVH. Additionally, the high global maximum dose in the lower IDL plans contributed to a heel region which was both higher and more extensive than the higher IDL plans.

For the curves in this portion of the investigation, the highest D_avg_ yielded the highest values of EUD_T_, EUD_R_, and TCP; there was a monotonic direct relationship observed between D_avg_ and these dosimetric parameters. For α = 0.20 Gy^−1^, the 40% IDL plan yielded a TCP of 91.8%, which is more than double (230%) the TCP observed with the 75% IDL plan. The EUD_T_ and EUD_R_ increased by 9.6% and 17.1%, respectively.

With the higher value of α = 0.23 Gy^−1^, the same trends were observed, but the differences were less mild; only a 12.9% increase in TCP changing from the 75% IDL plan to the 40% IDL plan. Note that the TCP values appear to be well within the plateau region of the TCP sigmoidal distribution. The EUD_T_ and EUD_R_ increased by 9.2% and 16.4%, respectively.

As with prior sections, the results of this portion of the investigation suggested that the shoulder value and shape were significant. However, this set of curves also demonstrated that the absolute value of the heel does carry significance.

## 4 DISCUSSION

### 4.A Radiobiological model limitations

It is well‐known that there is no single unique value for each radiobiological parameter — rather, it is realistically a range which is dependent on the population examined. Therefore, two values of α were used to generate EUD and TCP. The lower initial value of α = 0.20 Gy^−1^ was in a region of the cell‐survival curve with steep gradients, to increase sensitivity in uncovering trends. A second value of α = 0.23 Gy^−1^ was utilized to (a) substantiate the trends observed uncovered with the more sensitive α value and to (b) provide more realistic and familiar clinical TCP values associated with GK.^10^

As with all radiobiological models, the results should be framed with respect to the limitations of the assumed LQ model. While other TCP calculation formalisms do exist,^11^ the LQ model was purposely selected for this investigation for its simplicity to identify and quantify possible trends. In this study, our target volume was considered as a homogenous population of tumor cells with identical radiosensitivity. However, this assumption may affect the TCP calculations^12^, ^13^ and our group is currently investigating this effect.

TCP was chosen over EUD as the main parameter to study in that it provides a biological endpoint rather than a dose value. EUD remains a useful conceptual tool for the planning team by which the large dose range of GK can be recalculated in a more familiar homogeneous dose scheme. Note that EUD is formulated upon the survival fraction — the same parameter which is used to calculate TCP — thus, fortunately, it is likely that a treatment planning system would have both EUD and TCP available.

### 4.B Overall trends

Many of the observations from our study are novel because of the uniquely large range of dose levels characteristic to GK treatment planning. While planners using conventional (1.8 Gy–2 Gy) fractionation typically encounter global maximums of only ~110%, standard radiosurgery plans typically contain maximums of ~135%, and GK radiosurgery plans typically contain global maximums of 200%.^1^, ^14^, ^15^ Within the expanded dose axis of the cDVH, GK plans tended to follow a general characteristic shape with the following three general regions: a shoulder, a fall‐off region, and a heel.

The first two sets of curves had similar minimum and maximum doses. In these curves, the extent of the shoulder region was shown to inordinately influence the magnitude of the TCP. This trend aligned with the radiobiological interpretation of the TCP calculation; cells receiving the lowest dose (near prescription dose and/or shoulder region) influence the TCP more than those cells which receive the highest doses (heel region). Thus, if presented with two plans with otherwise similar minimum and maximum doses, our investigation demonstrated that the plan with the broader shoulder region would produce the superior TCP.

The last set of curves introduced a variable maximum dose. These curves revealed that given two plans with otherwise similar minimum doses and shoulder shapes, the plan with the higher maximum dose would produce the superior TCP. In the curves created using the SWS technique, a higher maximum dose resulted in a smaller heel region on the cDVH, but this was counteracted by larger values throughout the remainder of each curve.

As the prescription isodose line was increased, the maximum dose was closer to prescription dose and the overall dose heterogeneity was eliminated. As a theoretical exercise, if an isodose could actually be increased further to 100%, the result would be a completely uniform delivery (i.e. EUD = prescription dose) and the cDVH would be a step‐function curve as in curve E of Fig. 1. This was earlier already shown to have zero TCP assuming an 18 Gy prescription as in Table 1. Again, this would be a purely theoretical result in GK, as a flat beam profile is unachievable without some type of flattening filter. However, these results imply that there may be an upper limit on the utility of higher isodose lines in GK. This is currently being investigated by our group.

The flexibility of the delivery system results in a myriad of dose distributions for the planning team to choose from. When selecting the plan to be delivered, our study established that the highest average dose was not necessarily correlated with largest TCP. On the other hand, both EUD metrics (EUD_T_ and EUD_R_) were shown to correlate with the TCP.

### 4.C Practical issues: multiple‐shot 4 mm vs single‐shot large collimator

Plans composed of multiple smaller 4 mm shots had distinguishable waves on the boundary of the PIV surface, especially when directly compared with the smooth circular distributions of a single‐shot of larger collimator. Thus, in order to account for underdosages from a wave, the PIV using the 4mm collimator was slightly enlarged compared to the larger collimator.

The enlarged PIV ensured a larger dose to the PIV near the prescription dose. This resulted in a larger shoulder in the cDVH than the coverage of a single larger collimator, which was demonstrated to have a significant effect on TCP. In addition, the slightly enlarged PIV resulted in a geometry which would be more robust to slight shifts in treatment delivery, which was investigated by prior studies.^16^

While the immediate bordering regions had higher dosages, after moving away 2–3 mm from the PIV, there was a dramatic drop in dose, as this was beyond the geometric outline of the 4mm collimator shot. Note that the current cDVH target structure was defined as the PIV with no margin. Thus, if the target was redefined with a 1mm margin, this would shift the shoulder of the cDVH to the right significantly.

### 4.D Delivery time efficiency

The clinical utility of GK can be limited by the protracted delivery times compared with the dose rates observed with other treatment modalities such as flattening filter‐free linear accelerators and/or CyberKnife.^1^, ^14^, ^15^ Indeed, prior studies by Johnson et al. and Wright et al. have utilized delivery time as a metric for plan optimization.^9^, ^17^ As observed with prior studies, delivery times were protracted significantly as plans moved toward smaller collimators due to geometric effect as well as the loss of output factor.

In the second dataset, this investigation demonstrated that using the 4mm collimator shots to cover a 16mm PIV resulted in a nearly unmanageable 130.3 min, compared with the 10.2 min when using a 16mm collimator. Note that these times are scaled to a 3.5 Gy/min dose rate, which is only available immediately after source exchange, and some institutions operate until sources are around one half‐life in age,^18^ thus effectively doubling these delivery times.

For large‐volume GK institutions, it is not uncommon to treat patients with 10+ target sites,^19^ which would further intensify the single PIV differences in overall treatment delivery time. In addition to reducing the clinical viability of a plan, there have been previous studies which have shown identical plans have been shown to produce lower biologically effective dose by 11.7% for GK deliveries as short as 30minute delivery times based upon intrafraction repair.^18^ In other delivery sites, such as the prostate, the effects of intrafraction repair and source decay have been shown to reduce the BED by as much as 36%.^20^

### 4.E Clinical impact and notes

By associating TCP and EUD with simple features within the cDVH, the general trends from this study may be integrated into GK treatment planning without relying upon the separate third‐party analysis software normally required for radiobiological calculations. This visual method expedited treatment planning by allowing prompt evaluation which also circumvented repeated data transfer between GK and third‐party analysis software. The efficient usage of time can be especially sensitive for GK clinics which perform frame‐placement, imaging, treatment planning, and delivery all within a timespan of a few hours.

Our investigation was performed exclusively for the large dose ranges ubiquitous with GK. Therefore, while the visual method can be utilized in GK, the reader is reminded that the same trends among dose–volume characteristics, TCP, and EUD may not necessarily be observed with different dose ranges. In general, as the overall dose range becomes narrower, the heel region is expected to have a larger impact. Users should be especially vigilant in applying our observed trends in GK to conventional fractionation, where the dose range is much smaller.

## 5 CONCLUSIONS

This investigation used two separate radiobiological parameter sets to identify general trends among TCP, EUD, and dose‐volume characteristics by examining basic features represented with a cDVH. Our investigation revealed general trends in the cDVH shape that can be used to quickly and reliably identify the superior plan among candidates. However, practical issues such as delivery time may require consideration by the planning team to choose the most clinically viable plan.

## CONFLICT(S) OF INTEREST

No conflict of interest.

## Appendix A

**Table A1:** Values for curve parameters, with curve E manually defined.

	Curve				
	A	B	C	D	E
D_min_ [Gy]	18.0	18.0	18.0	18.0	18.0
D_max_ [Gy]	36.0	36.0	36.0	36.0	36.0
D_1_ [Gy]	21.6	21.6	21.6	21.6	–
D_mid_ [Gy]	25.2	25.2	25.2	25.2	–
D_2_ [Gy]	28.8	28.8	28.8	28.8	–
h_1_	0.4	0.65	0.1	0.1	–
h_2_	0.2	0.8	0.15	0.8	–

Abbreviations: D_min_, Minimum dose, D_max_, maximum dose.
